# Exploring the association between interleukin-1β and its interacting proteins in Alzheimer’s disease

**DOI:** 10.3892/mmr.2015.3183

**Published:** 2015-01-12

**Authors:** LUSHUANG XIE, YU LAI, FANG LEI, SUJUAN LIU, RAN LIU, TINGHUA WANG

**Affiliations:** 1 Department of Histology and Neurobiology, West China School of Preclinical and Forensic Medicine, Sichuan University, Chengdu, Sichuan 610041, P.R. China; 2 Department of Histology, Chengdu University of Traditional Chinese Medicine, Chengdu, Sichuan 610072, P.R. China; 3 Department of Physiology, West China School of Preclinical and Forensic Medicine, Sichuan University, Chengdu, Sichuan 610041, P.R. China

**Keywords:** Alzheimer’s disease, interleukin-1β

## Abstract

Alzheimer’s disease (AD) is an age-associated progressive neurodegenerative disorder which is of clinical concern. The association between the nervous and immune system is defined as an neuroimmunological theory that supports a model of pathology or treatment for AD. Interleukin (IL)-1β has a pro-inflammatory function in AD; however, the mechanism of its dysregulation in AD remains unknown. It is therefore of significance to understand the molecular mechanisms of IL-1β and how it may regulate AD. Proteins, which have been previously reported to be associated with IL-1β in AD, have been used in the present study as nodes to illustrate a net of protein interaction in Cytoscape. The Kyoto Encyclopedia of Genes and Genomes was used to further analyze the association of these proteins with the pathology of AD. The present study identified and subsequently compared two AD and six IL-1β pathways with the network produced in Cytoscape. The present study identified important mechanisms in the pathology of AD and constructed two novel networks using Cytoscape.

## Introduction

Alzheimer’s disease (AD) is an age-associated progressive neurodegenerative disorder that is characterized by the presence of senile plaques, neurofibrillary tangles and loss of synapses (1,2). This disease not only endangers health and is detrimental to the life of patients with AD, but additionally imposes a considerable burden on families, carers and society (3). Effective treatment for AD has been an ongoing challenge to clinical workers and researchers (4,5). Current therapeutic interventions act to relieve symptoms and delay the progression of AD; however, the development of novel strategies is required to improve treatment outcomes (6,7).

Neuroimmunological theory investigates the association between the nervous and immune system and involves numerous neuronal diseases, including Alzheimer’s, Parkinson’s and Huntington’s disease (8,9). Analyzing the association between the nervous and immune system may therefore generate valuable insight leading to novel therapeutic approaches (10). Cytokines are small molecular polypeptides secreted by immunocytes and other associated immunological cells, including astrocytes, that mediate and regulate the immune and inflammatory response (11,12). Regulation of the immune response is associated with the pathogenesis of AD (13). Interleukin-1 beta (IL-1β) is a cytokine that has an important modulatory effect on the pathology of AD. IL-1β functions as a pro-inflammatory cytokine, and an increase in IL-1 expression has been associated with AD (14). Increased serum levels of IL-1β are used as a stage marker of the ongoing brain neurodegeneration in the continuum between normal ageing and AD (15). Although a direct association between IL-1β and AD has been identified (16,17), the most accepted view supports that the pro-inflammatory effects of IL-1β promote a trend towards disease deterioration, but this process involves numerous factors and pathways. Few studies have further investigated the association between IL-1β and other proteins and pathways involved in the pathogenesis of AD. Synthetic analysis of the relationship between these additional factors and IL-1β in AD may facilitate the identification of the molecular mechanisms of IL-1β function in the pathogenesis of AD (18,19).

Cytoscape (http://www.cytoscape.org/) (20) is an open source software project for integrating biomolecular interaction networks with high-throughput expression data and other molecular states into a unified conceptual framework. This software has been extensively used by researchers to investigate biological domains, the genome, proteome and metabonomics. A large database of biological interactions, including protein-protein and protein-DNA, are displayed by the software in graphical format, displaying key nodes and end-points (20–22). The Kyoto Encyclopedia of Genes and Genomes (KEGG; www.kegg.jp) is a database resource for systematic analysis of gene function, in terms of the networks of genes and molecules. The central feature of the KEGG resource is the PATHWAY database that consists of graphical diagrams of biochemical pathways, including most of the known metabolic pathways and some of the known regulatory pathways (23,24).

Two of the referenced pathways in KEGG are associated with AD. One is a direct AD pathway and the other is a WNT signaling pathway. Several factors which are associated with IL-1β in AD have been identified in some of the pathways in the KEGG database. By using Cytoscape, this study has identified the associations between IL-1β and its associated proteins in AD. By comparing these networks produced by Cytoscape with the KEGG pathways, the mechanism and function of IL-1β in AD was investigated.

## Materials and methods

### Data mining

The factors of interest were selected based on data mining in the published literature. Factors were selected based upon associations with IL-1β in AD (25–27). The factor pool was constructed and is shown in Table I. Additional factors were constituted from additional factor pools which included proteins which have not previously been described to affect IL-1β but which have a moderate association (Table II).

### Data reduction

Cytoscape provides a convenient procedure to construct functional interaction nets. Data were uploaded through one of the supported formats, including Microsoft EXCEL. Data was therefore constructed in tabular form in EXCEL, two columns represented nodes and one column represented edges. The attributes of these nodes and edges were compiled using Notebook (Smart Software, Calgary, AB, Canada). The attributes of the nodes were associated with these classifications of factors and the ones of edges were associated with an interaction between these nodes annotated as upregulating or downregulating. The map nodes and edges, attributes and default visual properties for all elements were therefore specified.

### Construction of networks using Cytoscape

The data were imported into Cytoscape through the ‘IMPORT NETWORK FROM TABLE’ function, and the network of associated factors with IL-1β in AD were generated automatically, only defining the interaction of the list in EXCEL.

### KEGG searches and reconstruction of associated images by Cytoscape

In order to further determine the affected pathways in AD through IL-1β, search terms including ‘Alzheimer’s disease’ or ‘IL-1β’ were used as keywords in the KEGG pathway analysis. Following this, certain pathways, which were identified to be associated with this research, were redrawn in Cytoscape in order to better compare these factors.

## Results

### Interaction nets created in Cytoscape

**Figure 1:**
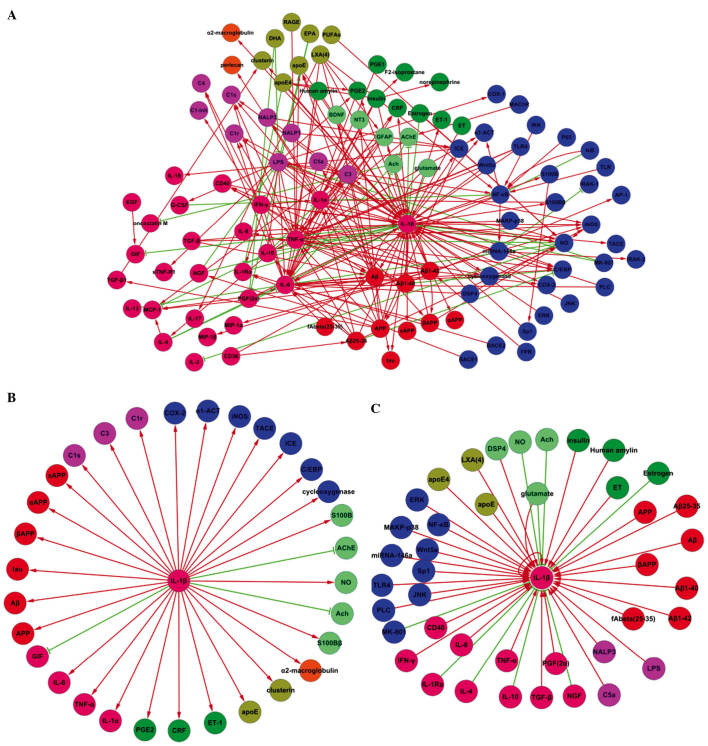
Association between IL-1β and other factors in AD. (A) Protein interaction map describing the responses implicated in the association between IL-1β and other factors. Increases are reflected as straight arrows, shown as red lines and decreases are reflected as inverted ‘T’ shapes, shown with green lines. (B) A protein interaction map describing the responses triggered in the factors affected by IL-1β in AD. (C) A protein interaction map describing the responses triggered in the factors affected by IL-1β in AD. The following colors were used in creating the cytoscape nets: Pink, cytokines; green, hormones, blue, signal factors; orange, globulin; purple, immune factors; blue-green, neurotransmitters; red, specific proteins with AD; brown, lipid. IL, interleukin; AD, Alzheimer’s disease.

Three interaction nets were generated. The factors were identified by querying the literature prior to collecting and constituting the interactional net between IL-1β and other factors involved in AD (Fig. 1A). Fig. 1B and C are subtypes of Fig. 1A. Fig. 1B includes the elements which could be affected by IL-1β in AD and Fig. 1C includes the proteins which could induce transformation of IL-1β in AD (see Fig. 1C).

### Analysis of the figures

#### Interaction net Fig. 1A

The association between IL-1β and other factors involved in Alzheimer’s disease is shown in Fig. 1A. This network included 105 nodes and 230 edges. Among these, 65 nodes and 75 edges were directly connected with the node of IL-1β. The edges consisted of 63 positive (straight arrow) and 12 negative correlations (inverted “T” shape). For the positive correlations, 35 of the edges represented that IL-1β would be affected by other factors, whereas 28 edges indicated that IL-1β would affect other factors. This applied to the negative correlations, where nine edges were inhibited and three edges were inhibiting.

The nets indicated that IL-1β is associated with numerous other factors involved in the pathology of AD. Several of these associated factors were significantly correlated with AD, including forms of beta amyloid (Aβ) and its precursor, amyloid precursor protein (APP). These data indicated that IL-1β is a central immunological factor which influences the formation of Aβ and fibrous plaques.

Numerous cytokines have been observed to alter in function or expression in AD, including tumor necrosis factor (TNF), interferon (IFN) and complement component 3 (C3). This has demonstrated that the process of immunity is involved in the pathology of AD. Furthermore, some of these factors are correlated with IL-1β, which may prove to be a central factor in AD-associated immunological reactions.

The pathology of AD is often comorbid with hormone disorders, thus the neuroendocrine system is additionally considered to participate in the pathology of AD. Hormonal secretions have been observed to be interrelated with IL-1β and the nervous, immune and the endocrine systems, which contribute to the pathology of AD.

The expression and accumulation of starch in the nervous system due to AD lesions has an effect on neuronal activity. Numerous neurotransmitters have been shown to be involved in this pathology of AD, in which certain aspects have been correlated with IL-1β.

The activation or inhibition of signaling pathways maintains the determination of gene expression and protein synthesis. IL-1β is dependent upon multiple pathways in order to exert effects on the pathology of AD. The interaction net created in Fig. 1A indicated that various signaling molecules may be associated with IL-1β, thus forming a signaling pathway between IL-1β and AD.

#### Interaction net Fig. 1B

The net shown in Fig. 1B was composed of 31 edges and 32 nodes. These represent factors that were increased or decreased by IL-1β. Of the 32 edges, 28 were upregulated and three were downregulated.

The interaction net indicated that IL-1β could promote the secretion of Aβ, which originates from APP cleavage products, including αAPP, βAPP, sAPP. IL-1β may therefore induce Aβ assembly through the upregulation of APP. Aggregates of Aβ in the central nervous system (CNS), including the hippocampus, would lead to neuronal toxicity, synapse damage and neuronal apoptosis.

The interaction net indicated that numerous inflammatory mediators may contribute to the pathology of AD. IL-1β acts as an inflammatory mediator that drives the secretion of other inflammatory factors, including IL-6, tumor necrosis factor (TNF)-α and complement C1s subcomponent (C1s), complement C1r subcomponent (C1r) and C3. The activity of astrocytes and microglia in the CNS has been previously observed in AD (28), which supports that neuroimmunomodulation participates in the process of AD. IL-1β, IL-6, TNF-α as well as complements C1 and C3 are pivotal factors in inflammation. The interaction net indicated that IL-1β can upregulate these inflammatory factors, thereby aggregating inflammation, which results in the generation of adverse conditions for immunological disorders of AD.

Neurological diseases are often accompanied by deterioration of the endocrine system (29). It has been reported that low levels of estrogen may be one cause of AD, which is prevalent in females following the menopause (30). The interaction net identified that IL-1β may upregulate the internal secretions of factors including corticotropin-releasing factor (CRF), prostaglandin E2 (PGE2) and endothelin (ET)-1. An increase in estrogen levels may change the cerebral blood supply and metabolism and result in abnormal neuronal viability.

According to the interaction map in Fig. 1B, IL-1β may affect the secretion of various neurotransmitters. IL-1β could enhance nitric oxide (NO) and S100B, which cause neuronal damage, whilst suppressing acetylcholin (ACh), which usually acts to protect neurons. It may be concluded that IL-1β expression has a disadvantageous function in AD, inducing neuronal loss and suppression of protective factors.

Pathway analysis was conducted to determine the influence of IL-1β and the affected signaling molecules in the interaction map. Fig. 1B shows that IL-1β may impact the production of prostaglandin-endoperoxide synthase 2 (COX-2), thus functioning in the respiratory chain and disturbing mitochondrial activity. Disturbance of mitochondrial function could be attributed to disturbance of neural structure and function, which ultimately results in apoptosis.

#### Interaction net Fig. 1C

The interaction map in Fig. 1C is composed of 43 edges and 42 nodes, which represent the factors that increase or decrease IL-1β expression. With the exception of eight nodes, 34 nodes including IL-1β itself, could upregulate IL-1β. Of the factors identified, glutamate was shown to both activate and inhibit IL-1β, thus the total number of nodes that could upregulate IL-1β was 35. These data showed that the majority of factors in AD may be attributed to the activation of immune cells, including monocytes, lymphocytes, astroglia and microglia in the CNS, to secret IL-1β. High levels of IL-1β would aggravate the inflammatory damage caused to the nervous system.

The interaction map indicated that Aβ and its degradation products are able to initiate the production of IL-1β. Accumulation of Aβ and the formation of fibrous plaques are typical characteristics of AD. The induced secretion of IL-1β by these products can aggravate the pathological changes that occur in AD. The secretion of various inflammatory substances, including cytokines IFN-γ, TNF-α and factors such as lipopolysaccharide (LPS), NACHT leucine-rich repeat protein (NALP3) and complement C5a, is a major component of the pathology of AD. An increase in these substances is associated with the production of IL-1β by immune cells, which stimulates an inflammatory reaction, thus leading to inflammation of the neuron. Lesions in the CNS develop cellular hypoxia and changes to the cytoskeleton, which further induce apoptosis. Despite the existence of numerous negative factors, protective elements, including IL-4 and IL-10, could inhibit IL-1β and prevent further pathological damage.

It is known that the absence of insulin can induce diabetes. Conversely, high levels of insulin have emerged to be able to upregulate IL-1β, and thus may be associated with AD. Estrogen is considered to be a protective factor which may prevent the occurrence of AD through inhibition of IL-1β. It has been shown that the immune system affects the CNS following endocrinium influences on the immune system. This evidence follows a review of what is known about the association between the nervous, immune and endocrine systems, which is a major focus in the medical treatment of AD (31). Accordingly, this interaction net illustrates that few neurotransmitters are associated with IL-1β, including ACh, which may be a protective factor in AD. There is conflicting evidence regarding the effects of glutamate, suggesting it could therefore increase or decrease IL-1β expression.

Signaling molecules, including the toll-like receptor (TLR)4 receptor for the TOLL signaling pathway and mitogen-activated protein kinase (MAPK)-P38 in the MAPK pathway, have been observed to affect IL-1β. These molecules contribute to induce the gene expression of IL-1β, therefore resulting in aggravation of inflammation of the CNS.

### Pathways collected from KEGG analysis

**Figure 2:**
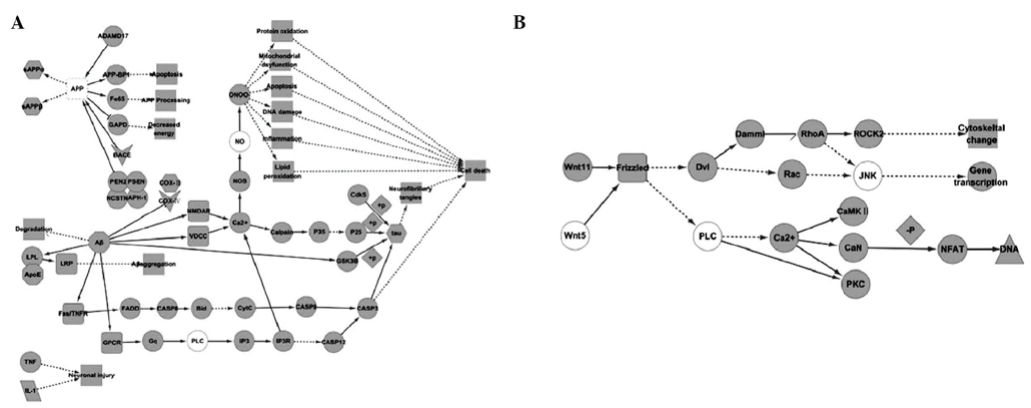
Kyoto Encyclopedia of Genes and Genomes pathway created using ‘Alzheimer’ as a key word. (A) Molecular map describing the responses triggered in the Alzheimer’s disease pathway. (B) Molecular map describing responses triggered in the WNT signaling pathway. Activation is reflected as a straight arrow and inhibition is reflected as an inverse ‘T’ shape. Indirect activation is reflected as a dotted arrow and indirect inhibition is reflected as a dotted inverted ‘T’ shape. Dissociation is reflected as equal lines and a missing interaction is reflected as a half arrow. The round-edged rectangle represents a membrane receptor and diamond shapes represent phosphorylation. The ‘V’ shape represents similarities with factors in Table II. The hexagon represents factors found in Fig. 1B, the ellipse with white color represents factors found in Fig. 1C and the octagon represents factors found in both Fig. 1B and C. Round-edged rectangles with white color indicate that both membrane receptor and be found in Fig. 1C and dotted round-edged rectangles indicated that both membrane receptor and be found in Table II. White dotted round-edged rectangles indicate that both membrane receptor and is found in Fig. 1B. Overlapping shapes represent a complex.

**Figure 3:**
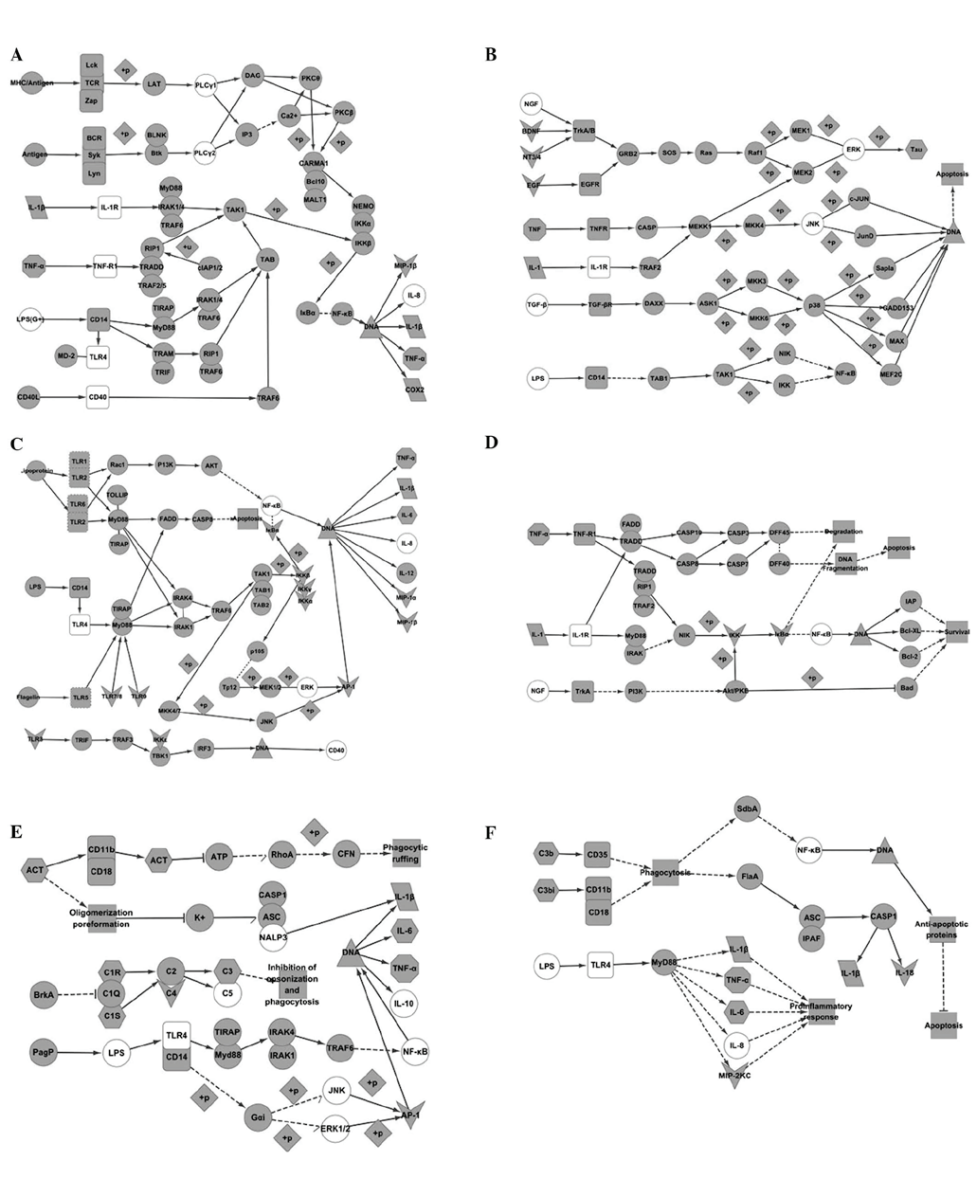
Kyoto Encyclopedia of Genes and Genomes pathway created using ‘IL-1β’ as a key word. (A) Molecular map describing the responses triggered in the NF-κB pathway. (B) Molecular map describing the responses triggered in the MAPK signaling pathway. (C) Molecular map describing the responses triggered in the Toll-like receptor signaling pathway. (D) Molecular map describing the responses triggered in the apoptosis signaling pathway. (E) Molecular map describing the responses triggered in the pertussis signaling pathway. (F) Molecular map describing the responses triggered in the legionellosis signaling pathway. IL, interleukin, NF-κB, nuclear factor kappa B; MAPK, mitogen-activated protein kinase. Activation is reflected as a straight arrow and inhibition is reflected as an inverse ‘T’ shape. Indirect activation is reflected as a dotted arrow and indirect inhibition is reflected as a dotted inverted ‘T’ shape. Dissociation is reflected as equal lines and a missing interaction is reflected as a half arrow. The round-edged rectangle represents a membrane receptor and diamond shapes represent phosphorylation. The ‘V’ shape represents similarities with factors in Table II. The hexagon represents factors found in Fig. 1B, the ellipse with white color represents factors found in Fig. 1C and the octagon represents factors found in both Fig. 1B and C. Round-edged rectangles with white color indicate that both membrane receptor and be found in Fig. 1C and dotted round-edged rectangles indicated that both membrane receptor and be found in Table II. White dotted round-edged rectangles indicate that both membrane receptor and is found in Fig. 1B. Overlapping shapes represent a complex.

Five results were obtained when using ‘Alzheimer’s disease’ as a key word. Among these results, map05010 and map04310 were objective pathways which could be used to compare with the interaction nets produced in Cytoscape (Fig. 2). Using ‘IL-1β’ as the key word, 27 results were obtained. These pathways included: i) The nuclear factor (NF)-κB signaling pathway, originating from map04064 in KEGG (Fig. 3A); ii) the MAPK signaling pathway, originating from map04010 in KEGG (Fig. 3B); iii) the TLR signaling pathway, originating from map04620 in KEGG (Fig. 3C); iv) apoptosis signaling pathway, originating from map04210 in KEGG (Fig. 3D); v) pertussis signaling pathway, originating from map05133 in KEGG (Fig. 3E); and vi) legionellosis signaling pathway, originating from map05134 in KEGG (Fig. 3F).

### Comparison of the AD pathway in KEGG with IL-1β and analysis of associated factors in AD

#### Comparison of Fig. 1B and Fig. 2A

Fig. 1B illustrates that IL-1β is able to upregulate NO. In addition, increases of NOS are driven by the accumulation of Ca^2+^ in the cytoplasm (Fig. 2A). Binding of NOS effectively causes secretion of NO-ONOO^−^, inducing protein oxidation, mitochondrial dysfunction, apoptosis, DNA damage, inflammation and lipid peroxidation. High levels of Ca^2+^ in the cytoplasm may be attributed to activating receptors, including FAS/TNFR, G protein-coupled receptor (GPCR), *N*-methyl-d-aspartate receptor and voltage-dependent calcium channels, whose ligands involve Aβ that can be increased by IL-1β. Accordingly, Fig. 1B shows that IL-1β can upregulate COX-2, which is effective in enhancing mitochondrial function. IL-1β can therefore cause an increase in Aβ, which would be associated with upregulation of COX-4 and Aβ-binding alcohol dehydrogenase. Mitochondrial dysfunction could therefore occur, which would increase reactive oxygen species production to induce cell death. It is widely recognized that APP is one of key factors in AD. Fig. 2A shows that the existence of APP has the potential to alter the expression levels of signaling molecules in the cytoplasm, including upregulation of Fe65, APP-binding protein 1 and downregulation of GAPD. These changes may lead to apoptosis and decreased energy production. This process is additionally associated with IL-1β, which may increase APP (Fig. 1B). In addition to Aβ and APP, IL-1β has been observed to upregulate apolipoprotein (Apo)E and Tau (Fig. 1B.) The former is connected with lipoprotein lipase, which can trigger LPR to cause aggregation of Aβ. The latter may cause formation of neurofibrillary tangles, which is a consequence of the activation of p25 and cyclin-dependent kinase (Cdk)5.

#### Comparison of Fig. 1C and Fig. 2A

Initiating factors, including Aβ, ApoE and APP (Fig. 2A) may increase IL-1β expression (Fig. 3C). The activation of neuronal signaling molecules has been associated with immunological functions, thus resulting in neuronal damage. The activation of Gq occurs through GPCR, which is the next molecule of oligomeric Aβ to which alterations would induce phospholipase C (PLC) to cause endoplasmic reticulum stress and cell injury by caspase (Casp)12. PLC was identified to be able to upregulate IL-1β (Fig. 1C).

#### Comparison of Fig. 1C and Fig. 2B

c-Jun N-terminal kinase (JNK) and PLC have been identified in the Cytoscape net and the KEGG pathway. G protein, which could activate PLC, would be expressed through signaling between Wnt5 and Frizzled, and Ca^2+^ influx that could trigger Ca^2+^/calmodulin-dependent protein kinase II, CaN and protein kinase C (PKC). These signaling molecules would dephosphorylate nuclear factor of activated T cells transcription factor, which would have effects on DNA expression (Fig. 2B). In addition, Wnt11 is responsible for the function of JNK through Rac or Ras homolog gene family, member A. Both PLC and JNK could increase the expression levels of IL-1β (Fig. 1C). The Wnt pathway not only participates in the pathology of AD, but can stimulate secretion of IL-1β, which in turn enhances neural immune damage.

### Comparison of the NF-κB signaling pathway with IL-1β and analysis of associated factors in AD

#### Comparison of Fig. 1B and Fig. 3A

The NF-κB signaling pathway is crucial for understanding the mechanism of inflammation. It was observed by analyzing Fig. 1B that IL-1β is both an initiation factor and objective factor. The connection of IL-1β and IL-1R initiates interleukin-1 receptor-associated kinase (IRAK)1/4-transforming growth factor β-activated kinase 1 (TAK1)-binding proteins (TAB)-inhibitor of nuclear factor kappa-B kinase subunit alpha (IKK) signaling, resulting in continuous phosphorylation of inhibitor of kappa B (IκB)α by IKK, which would expose the DNA binding site for NF-κB (p50/p65), ultimately inducing IL-1β, TNF-α and COX-2 expression. It was observed that IL-1β itself, TNF-α and COX-2 are upregulated by IL-1β in AD (Fig. 3A). It is therefore predicted that an inflammatory reaction in AD would be equivalent to the activation of the NF-κB signaling pathway.

#### Comparison of Fig. 1C and Fig. 3A

Factors, including IL-1β, IL-8, TNF-α, CD40, TLR4, PLC and LPS were observed in both the interaction net and KEGG pathway. Communication between TNF-α and TNF-R1 may activate TNF receptor type 1-associated DEATH domain protein (TRADD)/receptor-interacting serine/threonine protein (RIP1)/TNF receptor associated factor (TRAF)2/5, and the downstream factor RIP1 would stimulate TAK1. Finally, secretion of IL-1β would be induced by IKK-IκB-NF-κB. The function of TLR4 is possibly through CD14 activated by LPS, which would cause NF-κB stimulation of DNA through Toll-interleukin 1 receptor (TIR) domain-containing adapter protein (TIRAP)/MyD88 or translocating chain-associating membrane protein (TRAM)/TIR domain-containing adaptor protein inducing interferon β (TRIF). A connection between major histocompatibility complex/antigen and T-cell receptor also addresses the activation of NF-κB through PLC-PKC. CD40 and may affect NF-κB through TRAF6. Furthermore IL-8 is consistent with IL-1β in its secretion through the activation of NF-κB (see Fig. 3A). IL-1β may be upregulated by TNF-α, CD40, LPS and TLR4 in AD (Fig. 1C). This is evidence to support the involvement of the NF-κB signaling pathway in AD.

#### Comparison of Table II with Fig. 3A

Macrophage inflammatory protein (MIP)-1β is the conjunct factor shown in Table II and Fig. 3A. MIP-1β is secreted by the activation of NF-κB, which would be stimulated by IL-1β (Fig. 3A). Thus it is predicted that IL-1β may upregulate MIP-1β through the NF-κB signaling pathway in AD.

### Comparison of the MAPK signaling pathway with IL-1β and analysis of the associated factors in AD

#### Comparison of Fig. 1B and Fig. 3B

Two factors, TNF-α and Tau, were identified in both figures. The function can be initiated by a connection between IL-1, IL-1R and TAB1-TAK1, leading to mitogen-activated protein kinase kinase (MEK)1/MEK2-mediated phosphorylation of tau. Hyperphosphorylated tau is a competitor of tau protein, which occupies the sites of microtubules, which would damage the normal structure of the cytoskeleton and lead to apoptosis. TNF-α would stimulate Casp or TRAF2 in this pathway.

#### Comparison of Fig. 1C and Fig. 3B

Six factors were identified when comparing the two figures produced by Cytoscape and KEGG. These factors included TNF-α, TGF-β, JNK, extracellular-signal regulated kinase (ERK), LPS and nerve growth factor (NGF), which would upregulate IL-1β. However, all of these are causative factors in the MAPK pathway. It has been shown that TGF-β and TNF-α effect TAK1, which can phosphorylate NF-κB-inducing kinase NIK/IKK and NF-κB (Fig. 3B), leading to secretion of IL-1β (Fig. 3A). LPS would connect to CD14, which regulates NF-κB. It was identified that c-fos would be secreted when TrkA/TrkB is signaling with NGF or brain-derived neurotrophic factor (BDNF) (see Fig. 3B); however, an association has not been identified with NF-κB. Furthermore, ERK, a downstream factor of transforming tyrosine kinase protein (Trk)A/TrkB, would up-regulate IL-1β (Fig. 1C), thus inferring an association between this signaling pathway and AD. JNK would increase IL-1β (Fig. 1C), but the nexus with IL-1β (Fig. 3B) was not identified.

#### Comparison of Table II with Fig. 3B

It was identified that EGF, BDNF and neurotrophin (NT)-3 were included in Table II and the MAPK pathway, which take effects as ligands but do not affect IL-1β.

### Comparison of the TLR signaling pathway with IL-1β and analyzing associated factors in AD

#### Comparison of Fig. 1B and Fig. 3C

Two factors, IL-6 and TNF-α, were found in both the Cytoscape net and the KEGG pathway. Both factors are increased when the initiation of the TLR lead to the weakening of the transcription factors, AP-1 or interferon regulatory factor (IRF)5. This process is additionally required for expression of IL-1β. However when TLR1/TLR2 connect to their appropriate activators, NF-κB can function in this pathway through AKT. It is known that IL-1β can activate NF-κB, therefore this pathway may be one mechanism for upregulating IL-6 and TNF-α through IL-1β.

#### Comparison of Fig. 1C and Fig. 3C

Eight factors were identified when comparing the Cytoscape interaction net in Fig. 1C with the KEGG pathway in Fig. 3C. These factors included IL-8, TNF-α, CD40, NF-κB, TLR4, JNK, ERK and LPS. The secretion of IL-1β may be upregulated through AP-1 upon interaction between TLR4 with LPS. LPS activates TIRAP/MyD88, of which the downstream targets are TAB1/2/TAK1-mitogen-activated protein kinase kinase (MKK)33/4/6/7-p38/JNK or TAB1/2/TAK1-MKK3/4/6/7-IKK-ERK (Fig. 3C). IL-1β can be increased by LPS, TLR4, JNK and ERK (Fig. 1C). This has supported that the Toll-like signaling pathway, specifically AP-1 trigged by LPS-TLR4, could result in an imbalance of IL-1β in AD. This pathway predicts that IL-8, TNF-α and CD40 are only effectors through activating the TLR.

#### Comparison of Table II and Fig. 3C

Six factors were identified when comparing Table II and the Toll-like pathway (Fig. 3C), including MIP-1α, MIP-1β, TLR, IKK, IκB and AP-1. IL-1β could be produced when TLR4 is activated (Fig. 3C). It was therefore inferred that AP-1 would increase IL-1β expression during AD, through activation of the Toll-like signaling pathway. IκB can be activated by IKK, exposing the DNA binding site for NF-κB, which is able to promote expression of IL-1β. IκB and IKK are positive regulators of IL-1β in AD. However, MIP-1α and MIP-1β may be expressed when triggering the TLR in this pathway (Fig. 3C).

### Comparison of the apoptosis signaling pathway with IL-1β and analysis of associated factors in AD

#### Comparison of Fig. 1B and Fig. 3D

TNF-α was identified in both the Cytoscape interaction net (Fig. 1B) and the KEGG pathway (Fig. 3D). Both TNF-α and IL-1β are apoptotic factors in this pathway, with objective enzyme Casp3 activated through TRADD/Fas-associated protein with death domain (FADD)-Casp10/Casp8 signaling. This results in cleavage of the Casp substance. The activity of Casp3 is a typical change in AD (Fig. 2A), thus it could be inferred that TNF-α and IL-1β may lead to apoptosis through the Casp3 pathway in AD.

#### Comparison of Fig. 1C and Fig. 3D

Three factors, including TNF-α, NF-κB and NGF, were identified when comparing Fig. 1C with Fig. 3D. In these pathways, NGF connects with TrkA which would in turn activate NF-κB through Akt/PKB. Other factors, including inhibitor of apoptosis (IAP), B-cell lymphoma-2 (Bcl-2) extra large protein (Bcl-XL) and Bcl-2 could inhibit this pathway. It is indicated that NGF may up-regulate IL-1β through this signaling pathway in AD.

#### Comparison of Table II and Fig. 3D

Only IKK was present in both Table II and the apoptosis pathway (Fig. 3D), which is associated with NF-κB.

### Comparison of the pertussis signaling pathway with IL-1β and analysis of associated factors in AD

#### Comparison of Fig. 1B and Fig. 3E

Pertussis is an infectious disease in which the pathological changes were analyzed through the signaling pathways shown in Fig. 1B and Fig. 3E. The pertussis signaling pathway was compared in order to further understand the molecular mechanisms contributing to AD. Six factors were identified in common between both figures, including IL-6, TNF-α, C3, C1r, C1s and actin assembly-inducing protein (ACT). The complement proteins C1r, C1s, and C3 would be activated upon triggering BrkA, which would inhibit opsonization and phagocytosis of macrophages. It was not identified whether these complements would affect IL-1β, IL-6 and TNF-α (Fig. 3E). Conversely, NF-κB would be weakened through cyclic adenosine monophosphate-p38 when activating ACT by cyanobacterial adenylate cyclase (CyaC) (Fig. 3E), which would ultimately result in the secretion of IL-1β, IL-6 and TNF-α.

#### Comparison of Fig. 1C and Fig. 3E

Nine factors were identified in common in Fig. 1C and Fig. 3E. These included IL-1β, IL-8, TNF-α, IL-10, TLR4, ERK, JNK, LPS, NALP3. The association between impact factors, including LPS, TLR4, ERK, JNK, and objective factors, including IL-10, IL-1β, IL-6 and TNF-α, are described in the preceding discussion. IL-1β would be expressed upon connection between Casp1 with ASC and NALP3, while this may be suppressed by high concentrations of K^+^ (Fig. 3E). This pathway also showed that IL-8 may inhibit the recruitment of immune cells and it is not conductive to inflammation elimination.

#### Comparison of Table II and Fig. 3E

Two factors, C4 and AP-1, were present in both Table II and the pertussis pathway. The function of AP-1 has been described in the Toll-like signaling pathway and C4 is a signaling molecule between C1 and C3/C5.

### Comparison of the legionellosis signaling pathway with IL-1β and analysis of associated factors in AD

#### Comparison of Fig. 1B and Fig. 3F

Legionellosis is another infectious disease which involves various factors that are associated with IL-1β in AD. These correlated elements include IL-6, TNF-α and C3. IL-1β would be secreted through NF-κB, which is downstream of Legk1 or SdbA, as well as through FlaA-Casp1. However, both signaling pathways are activated through a connection between C3 with CD35 or CD11b/CD18 (Fig. 3F). It could not be shown that C3 is upregulated by IL-1β in this pathway.

#### Comparison of Fig. 1C and Fig. 3F

Six factors, including IL-8, TNF-α, NF-κB, TLR4, LPS and IL-1β, were identified in both Fig. 1C and Fig. 3F. IL-1β, TNF-α and IL-8 would be upregulated by LPS and TLR4, which belong to the Toll-like receptor signaling pathway.

#### Comparison of Table II and Fig. 3F

Two factors, MIP and IL-18, were identified in both Table II and the legionellosis pathway. The function of MIP has been described in the Toll-like signaling pathway. IL-18 and IL-1β are produced by Casp1, which is directly stimulated by C3 (see Fig. 3F).

#### Integration of networks created by Cytoscape and KEGG

Through comparison of the networks created by Cytoscape and KEGG, the present study identified that certain factors may affect IL-1β in AD, which is contradictory to previous reports, which did not show that these factors up- or downregulated IL-1β. These factors included: i) MIP-1β, which is secreted by IL-1β; ii) IKK and IκB, which can both affect IL-1β through the NF-κB pathway; and iii) AP-1, which can increase the expression levels of IL-1β.

## Discussion

A growing body of evidence indicated that IL-1β expression is one of the earliest and most important neuropathological factors in numerous diseases of the nervous system, including AD (32,33). The present study therefore collected numerous factors which are known to be associated with IL-1β in AD and constructed an associated net using Cytoscape. Furthermore, pathways that are associated with AD or IL-1β were analyzed in order to determine the predominant functional mechanisms.

**Figure 4:**
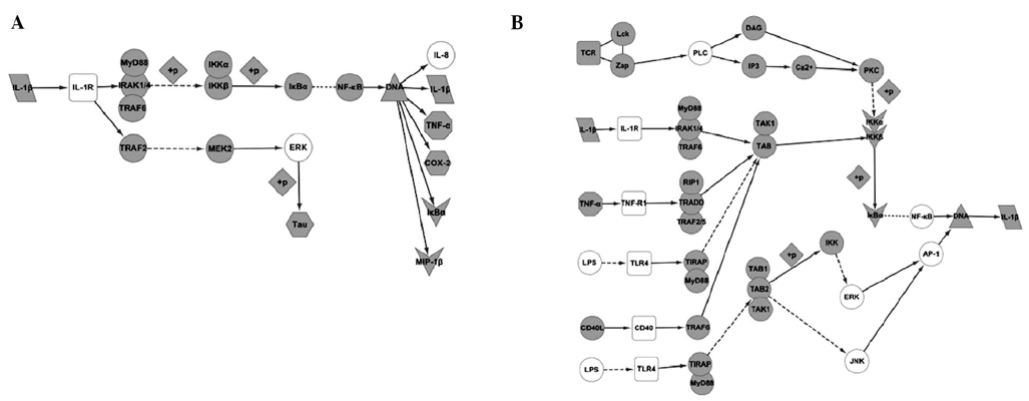
Functional mechanisms of the pathology of Alzheimer’s disease are associated with IL-1β. (A) Interaction map describing the signaling pathways triggered by IL-1β. (B) Interaction map describing the signaling pathway that induces changes to IL-1β in Alzheimer’s disease. IL, interleukin.

IL-1β was shown to connect with IL-1R, which would activate numerous pathways and subsequently lead to various pathological changes in AD. This would cause an energy metabolism disorder of the neurons and changes to the cytoskeleton. This would precipitate neuronal apoptosis and the formation of fibrous plaques. Certain important pathways would be employed in this change, including: i) the NF-κB signaling pathway, which would be activated when IL-1β is connecting with IL-1R. This would induce expression of IL-1β itself as well as TNF-α, Cox-2 and MIP-1β, and ii) the MAPK signaling pathway, with the main effect in this pathway being the secretion of Tau (Fig. 4A).

IL-1β is one of numerous effectors in AD, which would be upregulated by various signaling molecules to promote neuronal damage. These pathways include: i) the NF-κB signaling pathway - besides activation by IL-1β itself, the NF-κB pathway could be activated by TNF-α, LPS, PLC, CD40, TLR4 and TGF-β, to induce secretion of IL-1β. ii) The MAPK signaling pathway - factors in this pathway include NGF, BDNF, RAS and C-fos. iii) The Toll-like receptor signaling pathway - this pathway would be activated by LPS and TLR4, with JNK, ERK and AP-1 being affected by this activation. iv) The caspase signaling pathway, which would be activated by NGF (Fig. 4B).

In conclusion, the present study aimed to analyze the association between known factors and IL-1β in AD, and predict the mechanisms of action by comparing these factors with pathways generated in KEGG. It was identified that molecules may modulate IL-1β, or be modulated by IL-1β, in AD, in which IL-1β is the core factor in the inflammatory pathology. Numerous factors may increase IL-1β expression, thus leading to immunological damage. However, certain factors were identified to decrease IL-1β and therefore have protective effects on neuronal damage.

## Figures and Tables

**Table I:** Classification of factors associated with IL-1β in Alzheimer’s disease.

	Upregulating IL-1β	Upregulated by IL-1β	Downregulating IL-1β	Downregulated by IL-1β
Specific proteins with AD	Aβ, Aβ1–40, Aβ1–42, Aβ25–35, APP, fAbeta (25–34)	Aβ, APP, βAPP, sAPP, βAPP, αAPP, tau		
Cytokines	IL-1β, IL-8, TNF-α, IFN-γ, TGF-β, PGF (2α), CD40	IL-1β, IL-6, IL-1α, TNF-α	IL-4, IL-10, IL-1Ra, NGF	GIF
Other immune factors	C5a, LPS, NALP3	C3, C1s, C1r		
Neurotransmitters	Glutamate	ACh	ACh, AChE	
Signal factor	NF-κB, Wnt5a, TLR4, JNK, ERK, MAPK-p38, PLC, Sp1, miRNA-146a, NO, DSP4 S100B, α1-ACT	ICE, iNOS, TACE, COX-2, cyclooxygenase, C/EBP, NO, S100Bβ,	MK-801	
Hormone	insulin, Human amylin, ET	CRF, PGE2, ET-1	Estrogen	
Lipid	apoE, apoE4, LXA (4)	apoE, clusterin		
Globulin		α2-macroglobulin		

IL, interleukin; NF-κB, nuclear factor kappa B; IFN, interferon; TGF, transforming growth factor; APP, amyloid precursor protein. TNF, tumor necrosis factor; PGF, placental growth factor; LPS, lipopolysaccharide; TLR, toll-like receptor; ERK, extracellular signal-regulated kinase; MAPK, mitogen-activated protein kinase; PLC, phospholipase C; miRNA, microRNA; ACT, antichymotrypsin; apo, apolipoprotein; LXA, lipoxin A; NGF, nerve growth factor; ACh, acetylcholin; Cox-2, prostaglandin-endoperoxide synthase 2; NOS, nitric oxide synthase; TACE, tumor necrosis factor-alpha converting enzyme; C/EBP, ccaat enhancer binding proteins; CRF, corticotropin-releasing factor; PGE2, prostaglandin E2; ET, endothelin; C3, complement component 3; C1s, complement C1s subcomponent; C1r, complement C1r subcomponent.

**Table II:** Classification of other factors associated with IL-1β in Alzheimer’s disease.

Type	Factors
Cytokines	MCP-1, MIP-1β, MIP-1α, CD36, G-CSF, TGF-β1, EGF, IL-13, oncostatin M, IL-2, IL-18, sTNF-R1
Other immune factors	C4, C1-lnh, NALP1
Neurotransmitters	BDNF, NT-3
Signal factor	BACE1, BACE2, IRAK-1, IRAK-2, Cox-IV, TLR, IKK, IκB, P65, FPR, MAChR, AP-1
Hormone	PGE1, norepinephrine F2-isoprostane
Lipid	RAGE, DHA, EPA, PUFAs
Globulin	Perlecan

MCP, monocyte chemotactic protein; MIP, macrophage inflammatory protein; TGF, transforming growth factor; EGF, endothelial growth factor; G-CSF, granulocyte colony stimulating factor; IL, interleukin; TNF, tumor necrosis factor; NALP, NACHT leucine-rich repeat protein; BDNF, brain-derived neurotrophic factor; NT, neurotrophin; BACE, beta-secretase; IRAK, interleukin-1 receptor-associated kinase; Cox, cytochrome C oxidase; TLR, toll-like receptor; IKK, inhibitor of nuclear factor kappa-B kinase subunit alpha; IκB, inhibitor of kappa B; FPR, formyl peptide receptor; MAChR, muscarinic acetylcholine receptor; AP, activator protein; PGE, prostaglandin E; RAGE, receptor for advanced glycation end-products; DHA, docosahexaenoic acid; EPA, eicosapentaenoic acid; PUFA, polyunsaturated fatty acid.

## Abbreviations

AD: Alzheimer’s disease
IL: interleukin
KEGG: Kyoto Encyclopedia of Genes and Genomes
TNF-α: tumor necrosis factor-alpha
